# Long Non-coding RNAs: Regulators of Viral Infection and the Interferon Antiviral Response

**DOI:** 10.3389/fmicb.2018.01621

**Published:** 2018-07-19

**Authors:** Lipeng Qiu, Tao Wang, Qi Tang, Guohui Li, Peng Wu, Keping Chen

**Affiliations:** Institute of Life Sciences, Jiangsu University, Zhenjiang, China

**Keywords:** long non-coding RNA, interferon, antiviral response, viral infection, interferon-stimulated genes

## Abstract

Interferons (IFNs) are a family of cytokines providing a robust first line of host innate defense against pathogenic infection, and have now been part of the standard treatment for viral infection. However, IFN based therapy can best be described as modestly effective. Long non-coding RNAs (lncRNAs) are a novel class of non-protein-coding RNAs that are capable of regulating gene expression at different levels, including chromatin, transcription, post-transcription, and translation. Recently, lncRNAs are found to be deregulated upon viral infection or IFN treatment, and some of them can modulate viral infection in an IFN-dependent or -independent manner. Due to the crucial roles of lncRNAs in viral infection and the IFN antiviral response, the modulation of specific lncRNAs may be involved to increase the IFN antiviral response and improve the clinical result of IFN-based therapy. In this review, we summarize lncRNAs that are deregulated by viral infection, with special focus on the functions and underlying mechanisms of some essential lncRNAs, and discuss their roles in viral infection and the antiviral response of IFN.

## Introduction

Interferons (IFNs) are a family of cytokines providing a robust first line of host innate defense against pathogenic infection. Upon viral infection, IFNs are actively transcribed, which then induces the expression of various interferon stimulated genes (ISGs), establishing an antiviral state in the target cells (Borden et al., 2007). Currently, IFNs are attractive therapeutic options to control chronic virus infections. They are classified into three types: type I (IFN-α,-β, -𝜀, -κ, and -ω), II (IFN-γ), and III (IFN-λ1/IL-29, -λ2/IL-28A, -λ3/IL-28B). Type I IFNs, predominantly interferon-α (IFN-α) and IFN-β, have been part of the standard treatment for hepatitis B virus (HBV) and hepatitis C virus (HCV) infection, and play important roles in the initial stages of viral infection (Borden et al., 2007; Lin and Young, 2014). However, IFN based therapy can best be described as modestly effective. In a study of HBV infection, IFN based therapy only reached 33% HBV e Antigen (HBeAg) seroconversion [from HBeAg to HBV e antibody (anti-HBeAg)], with 25% of HBeAg positive patients achieving undetectable HBV DNA (Yuen and Lai, 2011). Therefore, great effort is needed to improve the clinical result of IFN-based therapy.

Long non-coding RNAs (lncRNAs), a novel class of non-protein-coding RNAs exceeding 200 nucleotides in length, are capable of regulating gene expression at different levels, including chromatin, transcription, post-transcription, and translation, and thus likely to be involved in innate immunity and viral replication (Imamura et al., 2014; Ouyang et al., 2014, 2016; Fortes and Morris, 2016; Valadkhan and Gunawardane, 2016). Recent studies demonstrated that, in response to viral infection or IFN, many lncRNAs were deregulated, and some of them impact on viral replication in an IFN-dependent or -independent manner; some viruses may hijack host lncRNAs to facilitate their replication and latency (Li et al., 2016; Ma et al., 2017; Wang P. et al., 2017). Due to the crucial roles of lncRNAs in viral infection and the IFN antiviral response, the modulation of specific lncRNAs may be involved to increase the antiviral response of IFN and improve the clinical result of IFN-based therapy. In this review, we summarize lncRNAs that are deregulated by viral infection and IFN, with special focus on the functions and underlying mechanisms of some essential lncRNAs, and discuss their roles in viral infection and the antiviral response of IFN.

## IFN Induction

**FIGURE 1:**
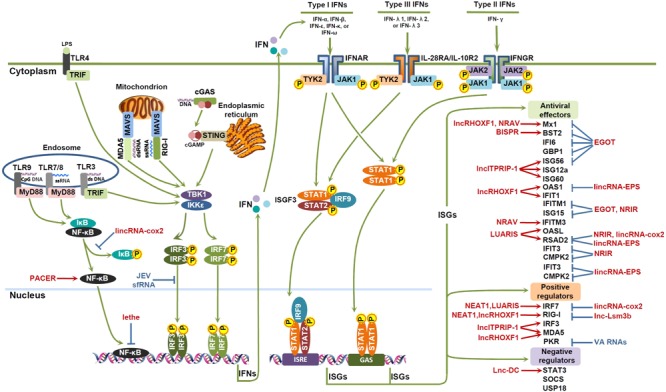
lncRNAs involved in the IFN antiviral response. Viral DNAs or RNAs are recognized by pattern recognition receptors (PRRs), such as retinoid acid inducible gene I (RIG)-I-like receptors (RLRs), Toll-like receptors (TLRs), and cyclic guanosine-monophosphate adenosine-monophosphate (cGAMP) synthase (cGAS). Subsequently, adaptor proteins, such as mitochondrial activator of virus signaling (MAVS), Toll-IL-1 receptor (TIR)-domain-containing adaptor inducing IFN-β (TRIF), myeloid differentiation primary response 88 (MyD88), and stimulator of IFN genes (STING), are activated, leading to the activation of interferon response factors (IRF) 3/7 or NF-κB. Phosphorylated dimers of IRF3/7 or NF-κB then translocate to the nucleus, bind to the promoters of target genes, and trigger the expression of IFNs. IFNs exerts their antiviral effects by binding to their corresponding receptors, and signaling through Janus kinase (JAK)-signal transducer and activator of transcription (STAT) pathway to induce the expression of IFN-stimulated genes (ISGs), which can function as antiviral effectors, positive regulators or negative regulators of the IFN pathway. Some host lncRNAs (red color) and viral lncRNAs (blue color) can modulate the activities of transcription factors, or the expression of ISGs, and thus impact on the IFN antiviral response.

Upon viral infection, viral features or pathogen-associated molecular patterns (PAMPs) are sensed by pattern recognition receptors (PRRs), such as retinoid acid inducible gene I (RIG)-I-like receptors (RLRs), Toll-like receptors (TLRs), and cyclic guanosine-monophosphate adenosine-monophosphate (cGAMP) synthase (cGAS). Subsequently, adaptor proteins, such as mitochondrial activator of virus signaling [MAVS, IFN-β promoter stimulator 1 (IPS-1), or Cardif], Toll-IL-1 receptor (TIR)-domain-containing adaptor inducing IFN-β (TRIF), myeloid differentiation primary response 88 (MyD88), and stimulator of IFN genes (STING), are activated, which then phosphorylate kinases NF-κB activator (TANK)-binding kinase-1 (TBK1) and inhibitor of κB (IκB) kinase (IKK) 𝜀, or the IKKα and IKKβ kinases, leading to the activation of interferon response factors (IRF) 3/7 or NF-κB. Phosphorylated dimers of IRF3/7 or NF-κB then translocate to the nucleus, bind to the promoters of target genes, and trigger the expression of IFNs (**Figure 1**; Schneider et al., 2014; Makris et al., 2017).

RLR family consists of three members, RIG-I (also known as DDX58), melanoma differentiation-associated gene 5 (MDA5, IFIH1), and laboratory of genetics and physiology 2 (LGP2), all of which are expressed in cytoplasm and specifically recognize viral RNA (Bruns and Horvath, 2015). Although they share similar structures, LGP2 lacks caspase activation and recruitment domain (CARD), and could not directly initiate downstream signaling like RIG-I and MDA5, while it was shown to participate in MDA5-mediated signaling (Bruns and Horvath, 2015). Upon binding of viral RNA, both of RIG-I and MDA5 were transported to the mitochondria, where they interact with the CARD of an adaptor protein MAVS and trigger the expression of type I IFNs (Liu S. et al., 2015). Viral nucleic acids can also be recognized by endosomal TLRs, including TLR-3 (ds RNA), TLR7/8 (ssRNA), and TLR-9 (unmethylated CpG DNA) (Lester and Li, 2014). Whist TLR3 interacts with TRIF and initiates TRIF-dependent signaling cascade, TLR7/8 and TLR-9 activate MyD88-dependent pathways (Lester and Li, 2014; Makris et al., 2017). Another TLR, cell surface expressed TLR4, may sense lipopolysaccharides (LPS) and induce the production of IFN-β through TRIF-mediated signaling (Fitzgerald et al., 2003). cGAS recognizes viral DNAs, and catalyzes the synthesis of the second messenger cGAMP, which binds to endoplasmic-reticulum-resident protein STING, resulting in the activation of IRF3 and induction of type I IFN (Ablasser et al., 2013; Sun et al., 2013; Wu et al., 2013). Other potential DNA sensors include DNA-dependent protein kinase (DNA-PK), IFN-γ-inducible protein 16 (IFI16, also known as AIM2, absent in melanoma 2), DNA-dependent activator of IFN-regulatory factors (DAI), and DExD/H-box helicase (DDX) 41, etc. (Mansur et al., 2014).

## IFN Signaling

Generally, IFNs may bind to their receptors, and exert antiviral effects in an autocrine/paracrine manner by signaling through Janus kinase (JAK)-signal transducer and activator of transcription (STAT) pathway to induce the expression of ISGs, establishing an antiviral state in the target cells (**Figure 1**; Borden et al., 2007). Except for the JAK-STAT pathway, IFNs also function by activating other STAT-independent pathways, such as mitogen-activated protein kinases (MAPKs) p38 and extracellular signal regulated kinases (ERKs), as well as the phosphatidylinositol 3-kinase (PI3K) pathway (Wang et al., 2004; Platanias, 2005).

Type I IFNs bind to a heterodimeric transmembrane receptor consisting of IFN-α receptor 1/2 (IFNAR1/IFNAR2), which then activating IFNAR1/IFNAR2-associated tyrosine kinase 2 (TYK2) and JAK1, resulting in the phosphorylation of STAT1 and STAT2. STAT1/STAT2, together with IRF9, forms the IFN-stimulated gene factor 3 (ISGF3), which translocates to the nucleus and binds to IFN-stimulated response elements (ISREs) in the promoters of ISGs to initiate their transcription. In addition to ISGF3, type I IFN also induce the formation of STAT1 homodimers, which directly translocate to the nucleus without assembly with IRF9, then bind to gamma-activated sequences (GASs) in the promoter of ISGs to stimulate gene transcription (Schneider et al., 2014). Type II IFNs bind to IFN-γ receptors 1/2 (IFNGR1/IFNGR2), which then activates their associated tyrosine kinases JAK1 and JAK2, leading to the phosphorylation of only STAT1 (Schroder et al., 2004). Phosphorylated STAT1 can form homodimers, which then translocate to the nucleus and bind to GAS in the promoter of ISGs to promote their transcription (Schroder et al., 2004). Type III IFNs, in contrast to type I IFNs, bind to a distinct receptor complex composed of IL-28RA and IL-10R2, while it triggers the same JAK-STAT signaling transduction cascades to activate gene transcription (Lopusna et al., 2013).

Although binding to different receptors, three types of IFNs signaling through similar JAK-STAT pathway. Types I and III IFNs activate the transcription of overlapping ISGs, while type II IFNs induces overlapping but distinct set of ISGs (Hertzog et al., 2011; Lopusna et al., 2013). These ISGs may function as antiviral factors, positive regulators or negative regulators of the IFN pathway (**Figure 1**). Antiviral factors impact on the different stages of the viral cycle. Among them, Myxovirus resistance (Mx) family of guanosine triphosphate (GTP)ases, IFN-inducible transmembrane (IFITM) and the tripartite motif (TRIM) family of proteins influence viral entry; IFN-induced protein with tetratricopeptide repeats (IFIT) family, the 2′–5′ oligoadenylate synthetase (OAS)-directed RNaseL pathway family proteins, protein kinase R (PKR), guanylate-binding proteins (GBPs), ISG15, ISG12a (IFI27), and G1P3 (IFI6, or 6–16) impact on viral replication, transcription, and translation; while radical s-adenosyl methionine domain-containing protein 2 (RSAD2, viperin) and bone marrow stromal cell antigen (BST)-2 (also known as tetherin) regulate viral assembly and release (Zhu and Liu, 2003; Zhu et al., 2003; Cheriyath et al., 2011; Sun et al., 2013; Liu et al., 2014; Yang et al., 2014; Xue et al., 2016; Chen et al., 2017). Positive regulators of IFN pathway include PRRs and signal transducing proteins, such as IRF3/7/9 and STAT1/2, and can be enhanced by IFN and reinforce the IFN response (Schroder et al., 2004). However, negative regulators, such as suppressor of cytokine signaling (SOCS), ubiquitin specific peptidase (USP) 18 and STAT3, may help IFN-induced cells to return to cellular homeostasis (Schroder et al., 2004; Wang et al., 2011).

## Deregulation of lncRNA Upon Viral Infection

Using lncRNA array and next-generation sequencing, aberrantly expressed lncRNAs in viral infection have been progressively unveiled. Various viruses, including influenza A virus (IAV), HCV, human immunodeficiency virus (HIV), herpes simplex virus (HSV), etc. have been reported to modulate the expression of multiple lncRNAs in host cells. One specific virus can result in the deregulation of multiple lncRNAs. For example, the infection of wild type or mutant IAV lacking NS1 (PR8ΔNS1, which failed to control IFN in infected cells) induces the expression of lncRNA EGOT (eosinophil granule ontogeny transcript), NEAT1 (Nuclear paraspeckle assembly transcript 1), BISPR (BST2 IFN-stimulated positive regulator, also known as LncBST2), VIN (virus inducible lincRNA), ISR (IFN-stimulated lncRNA)2 and ISR8, while it inhibits the expression of lncRNA NRAV (negative regulator of antiviral response, also known as DYNLL1AS1) (Barriocanal et al., 2014; Carnero et al., 2014, 2016; Imamura et al., 2014; Ouyang et al., 2014; Winterling et al., 2014). HCV infection enhances the expression of lncRNA BISPR, NRIR (negative regulator of interferon response), EGOT, ISR2, ISR8, GAS5 (growth arrest-specific 5), and lncITPRIP-1, etc. (Barriocanal et al., 2014; Carnero et al., 2014, 2016; Kambara et al., 2014; Qian et al., 2016; Xie et al., 2018). HIV infection promotes the expression of ISR2 and NEAT1, while it inhibits the expression of NRON [non-coding repressor of Nuclear Factor of Activated T cells (NFAT)] (Zhang et al., 2013; Carnero et al., 2014; Imam et al., 2015). Likely, one specific lncRNA can also be deregulated by the infection of different viruses. For instance, NEAT1 was reported to be up-regulated by IAV, HIV, HSV, and Hantaan virus (HTNV); ACOD1, a lncRNA identified by its nearest coding gene aconitate decarboxylase 1 (Acod1), can be increased by IAV, HSV and vascular stomatitis virus (VSV); while NRAV can be inhibited by the infection of IAV, HSV and Sendai virus (SeV) (Zhang et al., 2013; Imamura et al., 2014; Ouyang et al., 2014; Ma et al., 2017; Wang P. et al., 2017; Wang Z. et al., 2017). These results indicate that lncRNAs were differentially expressed in viral infection, and may be involved in viral replication and pathogenesis.

## Host lncRNAs as Pivotal Regulators of IFN Antiviral Response

### Host lncRNAs Modulate IFN Induction

NEAT1, an essential component for paraspeckle formation, may interact with mammalian *Drosophila melanogaster* behavior and human splicing (DBHS) proteins, including splicing factor proline- and glutamine-rich (SFPQ) protein, paraspeckle component 1 (PSPC1), and a non-POU domain containing, octamer-binding NONO/p54^nrb^ protein (Naganuma and Hirose, 2013). Recently, it was reported to be involved in cancer progression and the infection of several viruses, including HTNV, HSV-1, IAV, HIV, and KSHV (Zhang et al., 2013; Imamura et al., 2014; Idogawa et al., 2017; Ma et al., 2017; Wang Y. et al., 2017; Wang Z. et al., 2017; Chen et al., 2018). Among these viruses, NEAT1 modulates HTNV through impacting on IFN induction (Ma et al., 2017). In HTNV infected human umbilical vein endothelial cells (HUVECs), silencing NEAT1 by siRNA significantly accelerated, while over-expression of NEAT1 by NEAT1-expressing plasmid effectively inhibited, HTNV replication, viral-specific mRNA and nucleocapsid protein (NP) expression (Ma et al., 2017). Results from *in vivo* studies demonstrated similar inhibitory effect of NEAT1 on HTNV virus titers and NP expression, and also found decreased IFN-β production in serum and remarkably reduced body weight (Ma et al., 2017). Further mechanism studies revealed that NEAT1 may promote IFN responses by acting as a positive feedback for RIG-I signaling (Ma et al., 2017). By interacting with and relocating SFPQ from the promoter regions of RIG-I and DDX60 to paraspeckles, NEAT1 removed the transcriptional inhibitory effects of SFPQ on RIG-I and DDX60, resulting in increased expression of transcriptional factor IRF7, which in turn induced the expression of IFN and NEAT1 (Ma et al., 2017). Except for this, NEAT1 also forms a multi-subunit complex with HEXIM1, which may interact with cGAS sensor and its partner polyglutamine binding protein 1 (PQBP1), resulting in the release of paraspeckle proteins, recruitment of STING, and activation of IRF3, initiating the activation pathway leading to the production of type 1 IFN (Morchikh et al., 2017). These results indicate that NEAT1 has a critical role in the antiviral response of IFN through RIG-I signaling and cGAS-STING-IRF3 pathway.

Most recently, lnc-Lsm3b and lncITPRIP-1, two IFN-inducible lncRNAs, were reported to impact on viral infection and IFN production by modulating PRRs RIG-I and MDA5, respectively (Jiang et al., 2018; Xie et al., 2018). Lnc-Lsm3b, which is transcribed from *Lsm3* loci, could be up-regulated upon the infection of RNA viruses, such as VSV or SeV, as well as DNA virus HSV-1, and could also be stimulated by high concentration of type I IFNs and various TLR ligands (Jiang et al., 2018). Knocking down of lnc-Lsm3b using siRNA significantly increased the production of IFN-α and -β, resulting in decreased replication of VSV (Jiang et al., 2018). Mechanism studies revealed that in viral infected cells, lnc-Lsm3b may competitively bind with RIG-I monomer to restrict its conformational shift, thus preventing its binding with viral RNA, resulting the inactivation of RIG-I downstream signaling and termination of IFNs production (Jiang et al., 2018). By contrast, lncITPRIP-1, the genomic loci of which is near to inositol 1,4,5-trisphosphate receptor interacting protein (ITPRIP, also known as DNACR), was reported to be induced by multiple viruses, including HCV, VSV, SeV, and HSV, while over-expression of this lncRNA significantly inhibited HCV replication (Xie et al., 2018). Further studies revealed that lncITPRIP-1 can bind to the c-terminal of MDA5, increase the oligomerization of MDA5, and mediate MDA5-triggered production of IFNs (IFN-β, IL-28A, and IL-29) and ISGs (ISG12a, ISG56, and ISG60), leading to the suppression of HCV replication (Xie et al., 2018).

NeST (IFNG-AS1, or Tmevpg1), the genomic loci of which is located adjacent to the *IFN-γ* gene in both humans and mice, was reported to promote IFN-γ expression, and is associated with host’s persistence from Theiler’s virus (Vigneau et al., 2003; Gomez et al., 2013). Mechanism study revealed that NeST can bind to WD repeat-containing protein 5 (WDR5), a component of histone H3 lysine 4 (H3K4) methyltransferase complexes, resulting in enhanced H3K4 trimethylation (H3K4me3) at the IFN-γ locus, and leading to enhanced transcription of IFN-γ (Gomez et al., 2013).

Another lncRNA, lncRHOXF1, is specifically expressed in human trophectoderm cells. It was found to be significantly increased by the infection of SeV, while knocking down of this lncRNA greatly reduced the production of viral mRNAs and decreased expression of ISGs Mx1 and IFIH (Penkala et al., 2016). Further studies demonstrated that in human trophectoderm progenitor cells, depleting lncRHOXF1 using siRNAs resulted in significant increase of proteins in IFN induction pathway and ISGs, such as PRR MDA5 and RIG-I, IFN-β, as well as ISGs Mx1, OAS1, and IFIT1 (Penkala et al., 2016). These results indicate that lncRHOXF1 play a pivotal role in controlling SeV infection by modulating the IFN antiviral response.

### Host lncRNAs Interfere With ISGs Expression

NRAV, located on chromosome 12q24, was primarily found to be down-regulated in IAV-infected A549 cells, and could significantly affect the replication of IAV (Ouyang et al., 2014). Over-expression of NRAV by retroviral vectors significantly enhanced, whereas knocking down of this lncRNA by shRNA-based lentivectors greatly reduced, IAV replication (Ouyang et al., 2014). In *in vivo* studies, transgenic mice expressing human NRAV demonstrated greater replication of IAV and more severe inflammation in lungs compared with wide-type mice (Ouyang et al., 2014). By cDNA array screening and qRT-PCR validation, differentially expressed genes in NRAV over-expressing A549 cells infected with IAV were detected. The results showed that the expression of several critical ISGs, including IFIT2, IFIT3, IFITM3, OASL, and Mx1, were greatly reduced in NRAV over-expressing cells; while the expression of these ISGs were significantly increased in NRAV knocking down cells (Ouyang et al., 2014). Similar results were obtained in IAV infected NRAV transgenic mice (Ouyang et al., 2014). However, forced expression of these antiviral ISGs in NRAV over-expressing cells greatly reversed the effect of NRAV on the replication of IAV, suggesting that NRAV may function by regulating the expression of these ISGs during viral infection (Ouyang et al., 2014). Further mechanism studies revealed that NRAV over-expression significantly increased transcription activation marker histone 3 lysine 27 trimethylation (H3K27me3) enrichment at *mxA* gene locus, whereas NRAV knockdown greatly promoted transcription inhibition marker H3K4me3 enrichment and inhibited H3K27me3 enrichments at *mxA* and *ifitm3* transcription start sites (Ouyang et al., 2014). These data suggest that NRAV promotes viral replication by inhibiting the expression of ISGs such as MxA and IFITM3 via promoting H3K27me3 and reducing of H3K4me3.

LUARIS (lncRNA upregulator of antiviral response interferon signaling), also known as lncRNA #32, is an IFN down-regulated lncRNA widely expressed in various human tissues, and has been reported to inhibit the replication of EMCV, HBV, and HCV (Nishitsuji et al., 2016). In immortalized human hepatocytes (HuS cells), over-expressing of LUARIS greatly inhibited EMCV levels, while silencing of this lncRNA significantly enhanced viral titers (Nishitsuji et al., 2016). Results from cDNA microarray demonstrated that, at the absence of LUARIS, treatment of IFN-β in HuS cells significantly reduced the expression of many known ISGs and chemokines, including IRF7, OASL (2′–5′-oligoadenylate synthetase-like protein), RSAD2, CCL5 (Chemokine C-C motif ligand 5), CXCL (C-X-C motif chemokine ligand) 11/ITAC (interferon gamma-inducible T-cell alpha chemoattractant), and CXCL10/IP-10; while in LUARIS over-expressing cells, many of them were greatly increased (Nishitsuji et al., 2016). Similar results were found for anti-HBV and anti-HCV activities of IFN-β in human primary hepatocytes, indicating the inhibitory role of LUARIS on viral replication may associate with its regulation of ISGs and chemokines (Nishitsuji et al., 2016). Mechanism studies revealed that the function of LUARIS may associated with heterogeneous nuclear ribonucleoprotein (hnRNP) U and activating transcription factor 2 (ATF2) (Nishitsuji et al., 2016). HnRNPU, a protein previously reported to inhibit the replication of RNA viruses, was found to improve the stabilization of LUARIS, and thus impacted on the expression of IP-10 and RSAD2 (Pichlmair et al., 2012; Nishitsuji et al., 2016). In contrast, ATF2 may bind to the promoter region of ISGs and regulate their expression with the help of LUARIS (Nishitsuji et al., 2016). These data implied that LUARIS may be negatively regulated by IFN to prevent the possible inflammatory damage induced by excessive IFN response.

BISPR, the genomic loci of which near to BST2, was identified to be highly up-regulated by IFN-α2 or IFN-λ in a dose- and time-dependent manner, and can be significantly increased upon the infection of mutant viruses IAV (PR8ΔNS1) and VSVM51R, two of which failed to control IFN in infected cells (Garcia-Sastre et al., 1998; Terstegen et al., 2001; Garcia-Sastre, 2011; Barriocanal et al., 2014). Similar results were shown in HCV or HEV infected Huh7 cells and the liver of HCV-infected patients (Barriocanal et al., 2014; Paliwal et al., 2017). Bioinformatics analysis suggested that BISPR could share the same promoter with BST2 (Barriocanal et al., 2014). Furthermore, the expression of both BISPR and BST2 was STAT-dependent, and BISPR could positively regulate the expression of BST2 (Barriocanal et al., 2014). Based on the inhibitory effect of BST2 on virion secretion, BISPR may be involved in regulating viral infection partially by increasing the expression of antiviral protein BST2.

NRIR, also known as lncRNA-CMPK2, is located near the genomic loci ISG CMPK2 (cytodine/uridine monophosphate kinase 2) and RASD2 (Kambara et al., 2014). It was found to be stimulated by IFN-α or IFN-γ (Kambara et al., 2014). However, when the JAK-STAT pathway was suppressed by JAK inhibitor ruxolitinib or depleting of STAT2, the up-regulation of CMPK2 was abrogated, suggesting the direct stimulating role of IFN on its transcription (Kambara et al., 2014). Furthermore, in IFN-stimulated hepatocytes, knocking down of NRIR resulted in the transcriptional up-regulation of many ISGs, including CMPK2, RASD2, ISG15, CXCL10, IFIT3, and IFITM1, and also led to remarkable suppression of HCV replication (Kambara et al., 2014). Clinical results demonstrated that the expression of NRIR was up-regulated in liver tissues of chronic HCV infected patients which have active IFN response (Kambara et al., 2014). These data suggest that NRIR promotes HCV replication by negatively regulate the IFN antiviral response.

EGOT is another lncRNA that can be stimulated by IFN-α (Carnero et al., 2016). It was found to modulate the infection of different viruses, including HCV, IAV and Semliki forest virus (SFV), and also play important roles in several cancers, such as gastric cancer, glioma and renal cell carcinoma (Xu et al., 2015; Carnero et al., 2016; Jin et al., 2017; Peng et al., 2017; Wu et al., 2017). Upon HCV infection, viral RNA was sensed by RIG-I and PKR, then NF-κB was activated and bound to the promoter region of EGOT, leading to increased expression of EGOT (Carnero et al., 2016). In HCV-infected Huh7 cells, depletion of EGOT using gapmers greatly reduced HCV genomes, titer, core and NS3 proteins (Carnero et al., 2016). Similar results were also observed in SFV-infected Huh7 cells (Carnero et al., 2016). Mechanism studies revealed that in EGOT depleting cells with or without HCV infection, the expression of several ISGs, including GBP1, ISG15, Mx1, BST2, ISG56, IFI6, and IFITM1, was observed to be significantly increased, indicating that EGOT may promote viral replication by blocking the IFN antiviral response (Carnero et al., 2016).

### Miscellaneous Host lncRNAs Participate in the IFN Antiviral Response

Four lncRNAs, including IFN-stimulated lncRNA (ISR) 2, 8, 12 and lncISG15, can be stimulated by IFN and different viruses, and their genomic loci are near to ISGs GBP1, IRF1, IL-6, and ISG15, respectively (Barriocanal et al., 2014; Carnero et al., 2014). Upon the treatment of IFN, the expression of ISR2 (GBP1 pseudogene 1), ISR8 (AC116366.6), and ISR12 (LOC100506178) was significantly stimulated at early (6–12 h, ISR2, and 8) or later times (48–72 h, ISR12), mimicking that of their neighboring genes GBP1, IRF1, and IL-6 (Barriocanal et al., 2014). Bioinformatics analysis showed that STAT1 and 2 as well as IRF1 and 2 may bind to the promoter of ISR8 and impact on its transcription, while lncISG15 may share the same promoter with ISG15 (Barriocanal et al., 2014; Carnero et al., 2014). Results from virus infected cells and clinical samples showed that both ISR2 and ISR8 can be greatly up-regulated by HCV infection, while lncISG15 can be significantly enhanced by the infection of mutant IAV (PR8ΔNS1) and VSV (M51R), indicating their possible roles in viral infection and IFN response (Barriocanal et al., 2014; Carnero et al., 2014).

Two lncRNAs, lincRNA-cox-2 and lincRNA-EPS, can be modulated by TLR ligands, and the deregulation of these two lncRNAs influence the expression of some ISGs. LincRNA-cox2, also known as Ptgs2os2 [prostaglandin-endoperoxide synthase (PTGS) 2, opposite strand 2], can be induced by TLR ligands in a MyD88 and NF-κB dependent manner, while the up-regulation of this lncRNA significantly reduced the expression of some ISGs, such as *irf7* and *Rasd2* (Carpenter et al., 2013). Mechanism studies suggested that lincRNA-Cox-2 may interact with HnRNP-A/B and hnRNP-A2/B1 to regulate gene expression (Carpenter et al., 2013). On the contrary, lincRNA-EPS (erythroid prosurvival), can be suppressed by TLR ligands, and the reduction of lincRNA-EPS significantly increased the expression of ISGs ifit2, Rasd2, Oas1 and gbp5 by recruiting hnRNPL (Atianand et al., 2016).

Several lncRNAs, including PACER, NKILA, and lethe, may modulate the activity of NF-κB (Rapicavoli et al., 2013; Krawczyk and Emerson, 2014; Liu B. et al., 2015). *PACER* (p50-associated Cox2 extragenic RNA), also known as PTGS2-AS1, is located near the genomic loci of *COX-2* gene, and functions as a positive regulator of NF-κB by binding to the repressive NF-KB subunit p50 and promoting the formation of RelA-p50 heterodimer; NKILA (NF-κB-interacting lncRNA) inhibits NF-κB activity by binding to NF-κB/IκB and blocking the phosphorylation sites of IκB from IκB kinase (IKK) and thus preventing the degradation of IκB; while lethe suppress NF-κB activity by blocking the binding site of RelA to target genes and subsequent transcription (Rapicavoli et al., 2013; Krawczyk and Emerson, 2014; Liu B. et al., 2015).

Besides, lnc-DC (lnc-Dendritic cell lncRNA), also known as WFDC21P (WAP four-disulfide core domain 21, pseudogene), may impact on IFN response by modulating STAT3 (Wang et al., 2014). It can directly bind to STAT3 and prevent its dephosphorylation by SHP1, and thus activate STAT3-dependent transcription. In another report, lncRNA lethe (after the “river of forgetfulness” in Greek mythology) is reported to be stimulated by STAT3, and promote HCV infection and inhibit the expression of some ISGs, including PKR, OAS, and IRF1 (Xiong et al., 2015). However, in this report, incorrect primers for lncRNA lethe were used, so it is still arguable whether this lncRNA play a role in HCV infection.

## Virus-Encoded lncRNAs Impact on the IFN Antiviral Response

To counteract the IFN antiviral response, viruses have evolved different strategies to minimize IFN production and the activation of IFN signaling (Devasthanam, 2014; Chan and Gack, 2016; Schulz and Mossman, 2016). Interestingly, recent findings found that virus encoded lncRNAs may also participate in the antiviral response of IFN. For example, PAN RNA (polyadenylated nuclear RNA) from KSHV interacts with several virus- and host-encoded factors, including IRF4. During the lytic phase of KSHV infection, the expression of PAN RNA reduces the expression of IFNα, IFNγ, and ISG RNaseL; sfRNAs (subgenomic flavivirus RNAs) from several viruses, including Japanese encephalitis virus (JEV), dengue virus, and West Nile Virus (WNV), may antagonize the antiviral response of IFN by inhibiting the IFN signaling, the expression of IFN-β or specific ISGs; adenovirus virus-associated RNA (VA) target the ISG PKR to regulate the expression of ISGs (Rossetto and Pari, 2011; Schuessler et al., 2012; Chang et al., 2013; Bidet et al., 2014; Kondo et al., 2014). Interestingly, a chimeric lncRNA HBx-LINE1, which is generated by the integration of HBV into host cell genome, may attenuate the IFN antiviral response by inhibiting microRNA (miRNA)-122, a negative regulator of HBV replication which can be suppressed by IFN (Qiu et al., 2010; Chen et al., 2011; Wang et al., 2012; Hao et al., 2013; Lau et al., 2014).

## Host lncRNAs Participate in IFN-Independent Antiviral Response

Except for functioning IFN antiviral response by regulating IFN induction and ISG expression, lncRNAs can also regulate viral infection and replication in an IFN-independent manner, impacting on the transcription of viral genes, the translocation of viral transcripts, the function of viral proteins, even host cell metabolism. For example, NRON, which has been reported to enhance HIV gene expression in primary CD4 T cells, decreases the binding of NFAT and viral transcriptional activator Tat to the HIV-1 long terminal repeat (LTR) promoter region, resulting in reduced HIV-1 replication and viral protein expression; whereas lncRNA uc002yug.2 (linc01426) enhances HIV-1 viral replication, LTR activity, and the activation of latent HIV by up-regulating HIV Tat and suppressing RUNX 1b/1c, a transcription factor which can bind with HIV-1 LTR to inhibit HIV-1 replication (Cron et al., 2000; Willingham et al., 2005; Imam et al., 2015; Li et al., 2016; Mousseau and Valente, 2017; Huan et al., 2018). NEAT1 modulates the replication of different viruses by multiple mechanisms. Except for impacting on RIG-I signaling and cGAS-STING-IRF3 pathway, NEAT1 also influence the transcription of viral genes or translocation of viral transcripts. In response to HSV infection, NEAT1 and two other paraspeckle protein components, P54^nrb^ and PSPC1, may associate with HSV DNA. By doing so, PSPC1 may recruit STAT3 to paraspeckles and viral gene promoters, and thus facilitate HSV replication and protein expression (Wang Z. et al., 2017). In contrast, upon HIV infection, NEAT1 reduces nucleus-to-cytoplasm export of HIV-1 transcripts which containing *cis*-acting instability elements (INS), such as *gag/pol* and *env* RNAs, resulting in reduced HIV-1 replication (Zolotukhin et al., 2003; Kula et al., 2011; Yedavalli and Jeang, 2011; Zhang et al., 2013). Similarly, lncITPRIP-1 regulates HCV replication through both IFN-dependent and IFN-independent manners. Except for mediating MDA5-triggered production of IFNs and ISGs, lncITPRIP-1 also enhances the inhibitory effect of MDA5 on HCV replication by facilitating the binding of MDA5 to viral RNA (Xie et al., 2018). Another lncRNA, GAS5, reduces HCV replication by interacting with HCV protease NS3 protein to decoy its function (Qian et al., 2016). Interestingly, lncRNA-ACOD1 can promotes viral infection by modulating host cell metabolism. It directly interacts with metabolic enzyme glutamic-oxaloacetic transaminease (GOT2) in cytoplasm, resulting in increased catalytic activity of this enzyme, leading to enhanced production of key metabolites required for viral replication (Kotzin et al., 2017; Wang P. et al., 2017). Besides these, other lncRNAs, such as VIN (viral inducible lincRNA), also reported to modulate viral replication, while it did not change type I IFN response, the underlying mechanism of which is still unclear (Winterling et al., 2014).

## Conclusion and Future Perspectives

IFN-mediated innate antiviral response is the first line of immune defense against viral infection. In the past decade, lncRNAs have been demonstrated to control fundamental biological processes at the epigenetic, transcription and post-transcriptional levels, and the deregulation of lncRNAs contributes to immune response, including IFN-mediated antiviral response. In this review, we summarized the deregulated lncRNAs upon viral infection, with special focus on the functions and underlying mechanisms of some important lncRNAs, and discussed their roles in the antiviral response of IFN. The functions and mechanisms of action of some essential lncRNAs in viral infection and IFN antiviral response are summarized in **Figure 1** and **Table 1**.

**Table 1:** Essential lncRNAs involved in viral infection and IFN antiviral response.

lncRNAs	Description	Stimuli	Deregulation	Function on viral replication	Affected genes/proteins	Mechanisms of action	Reference
**IFN-DEPENDENT ANTIVIRAL EFFECT**		
NEAT1/VINC/ Linc00084	Nuclear paraspeckle assembly transcript 1; Virus inducible non-coding RNA	IAV, HSV, HIV, HTNV	↑	(-) HTNV	RIG-I, DDX60, IRF7, IFN-β	Promotes the expression of antiviral genes by interacting with and relocating SFPQ to increase the expression of RIG-1 and DDX60, leading to enhanced production of IFN-β.	Zhang et al., 2013; Imamura et al., 2014; Ma et al., 2017; Morchikh et al., 2017; Wang Z. et al., 2017
Lnc-Lsm3b	–	IFN-α, IFN-β, VSV, SeV, HSV	↑	(+) VSV	RIG-I, IFN-α, IFN-β	Inactivate the RIG-I function and IFN production by competitively binding RIG-I monomers to restrict its conformational shift, thus preventing its binding with viral RNA.	Jiang et al., 2018
LncITPRIP-1	–	IFN-α, HCV, VSV, SeV, HSV	↑	(-) HCV	MDA5, IRF3, IFN-β, IL-28A, IL-29, ISG12a, ISG56, ISG60	Binds to the c-terminal of MDA5, increase the oligomerization of MDA5, and mediates MDA5-triggered production of IFNs and ISGs.	Xie et al., 2018
NeST/IFNG-AS1/Tmevpg1	Nettoie Salmonella pas Theiler’s; IFNG antisense RNA 1; Theiler’s murine encephalitis virus persistence candidate gene 1	Theiler’s virus	↑	(+) Theiler’s virus	IFN-γ	Promotes the expression of IFN-γ by binding to WDR5 and enhancing H3K4me3 at the IFN-γ locus, increasing Theiler’s virus persistence.	Vigneau et al., 2003
LncRHOXF1/ RHOXF1P1	Rhox homeobox family member 1 pseudogene 1	SeV	↑	(-) SeV	RIG-I, MDA5, Mx1, OAS1,IFIT1, IFN-β	Promotes the expression of PRRs and ISGs.	Penkala et al., 2016
NRAV/DYNLL1-AS1	Negative regulator of antiviral response; DYNLL1-antisense RNA 1	IAV, SeV, HSV, MDRV	↓	(+) IAV	Mx1, IFITM3	Inhibits the transcription of MxA and IFITM3 genes by promoting H3K27me3 and inhibiting H3K4me3 on their promoters.	Ouyang et al., 2014
LUARIS/lncRNA #32	lncRNA upregulator of antiviral response interferon signaling	IFN-β	↓	(-) EMCV, HBV, HCV	IRF7, OASL, RSAD2	Facilitates the expression of ISGs by interacting with hnRNPU and ATF2.	Nishitsuji et al., 2016
BISPR/LncBST2	BST2 inteferon stimulated positive regulator	IFN-α2,IFN-λ, IAV(PR8ΔNS1), VSV(M51R), HCV, HEV	↑	(-) HEV	BST2/tetherin	Promotes the expression of BST2/tetherin.	Barriocanal et al., 2014; Paliwal et al., 2017
NRIR/lncRNA-CMPK2	Negative regulator of interferon response	IFN-α, IFN-λ, HCV	↑	(+) HCV	CMPK2, viperin, ISG15, IFIT3, IFITM1	Inhibits the expression of ISGs at transcriptional level.	Kambara et al., 2014
EGOT	Eosinophil granule ontogeny transcript	IFN-α2, IAV, HCV, SFV	↑	(+) HCV	GBP1, ISG15, Mx1, BST2, ISG56, IFI6 and IFITM1	Inhibits the expression of ISGs.	Carnero et al., 2016
**IFN-INDEPENDENT ANTIVIRAL EFFECT**
NRON	Non-coding repressor of NFAT (nuclear factor of activated T cells)	HIV	↓	(-) HIV	NFAT,HIV Tat	Inhibits HIV replication and viral protein expression by decreasing the abundance of NFAT and viral transcriptional activator Tat, reducing their binding to the HIV-1 LTR promoter region.	Imam et al., 2015; Li et al., 2016
uc002yug.2/ Linc01426	–	HIV	↑	(+) HIV	HIV Tat, RUNX 1b/1c	Enhances HIV-1 viral replication, LTR activity as well as the activation of latent HIV by up-regulating Tat protein expression and suppressing RUNX 1b/1c, a transcription factor which can bind with HIV-1 LTR to inhibit HIV-1 replication.	Huan et al., 2018
NEAT1/VINC/ Linc00084	Nuclear paraspeckle assembly transcript 1; Virus inducible non-coding RNA	IAV, HSV, HIV, HTNV	↑	(-) HIV	HIV INS-containing proteins	Inhibits HIV replication by decrease nucleus-to-cytoplasm export of INS-containing viral proteins.	Zhang et al., 2013
				(+) HSV	STAT3	Promotes HSV replication and protein expression by associating with HSV DNA together with P54nrb and PSPC1, leading to the recruitment of transcription factor STAT3 to viral gene promoters.	Wang Z. et al., 2017
LncITPRIP-1	–	IFN-α, HCV, VSV, SeV, HSV	↑	(-) HCV	MDA5	Promotes the inhibitory effect of MDA5 on HCV replication by facilitating the binding of MDA5 to viral RNA.	Xie et al., 2018
GAS5/SNHG2	Growth arrest specific 5; small nucleolar RNA host gene 2	HCV	↑	(-) HCV	HCV NS3	Inhibits HCV replication by interacting with HCV protease NS3 protein to decoy its function.	Qian et al., 2016
lncRNA-ACOD1/ LOC102637961	A lncRNA identified by its nearest coding gene Acod1, aconitate decarboxylase 1;	IAV, HSV, SeV, VSV, VACV	↑	(+) HSV, VSV, VACV	GOT2	Promotes viral infection and replication by interacting with GOT2 and increasing its catalytic activity to produce key metabolites required for viral replication.	Kotzin et al., 2017; Wang P. et al., 2017
VIN/Linc01191	Viral inducible lincRNA	IAV, VSV	↑	(+) IAV	Unknown	Unknown	Winterling et al., 2014

*IAV, influenza A virus; HSV, herpes simplex virus; HIV, human immunodeficiency virus; HTNV, hantaan virus; VSV, vesicular stomatitis virus; SeV, sendai virus; MDRV, Muscovy duck reovirus; EMCV, encephalomyocarditis virus; HBV, hepatitis B virus; HCV, hepatitis C virus; IAV(PR8ΔNS1), NS1 mutant PR8 virus which failed to control IFN signaling, belong to H1N1 genotype; VSV(M51R), M51R mutant VSV which failed to control IFN signaling; HEV, hepatitis E virus; VACV, Vaccinia virus.*

Except for impacting on IFN-mediated antiviral response, lncRNAs also modulate viral infection or replication by other mechanisms, such as regulating viral gene transcription, viral RNA translocation, viral protein function, and host cell metabolism. Interestingly, one lncRNA, NEAT1, has been reported to exert different effects on different viruses: it inhibits HTNV or HIV replication by activating IFN signaling or improving the translocation of INS-containing viral RNAs, while enhances HSV replication by facilitating the binding of STAT3 on the viral gene promoters. These indicate that different therapeutic strategies should be used to control different viruses. When host lncRNAs exert modulation on immune response, viruses have evolved to facilitate their survival and replication by regulating the expression of lncRNAs to influence different host pathways, suggesting the pivotal regulatory roles of lncRNAs in the interplay between host and viruses. Until now, only a small part of lncRNAs have been identified and characterized to participate in the IFN antiviral response, while the vast majority of uncharacterized lncRNAs remain to be further explored. More extensive studies are required to describe the precise profile of virus regulated lncRNAs and their functions in viral infection. Furthermore, the regulatory mechanisms of these lncRNAs by different viruses, as well as the underlying mechanisms of these lncRNAs in viral infection need to be fully elucidated. The investigation of the interaction between lncRNA and the IFN antiviral response may not only deepen our understanding of antiviral response, but also provide novel applications for better prognosis and antiviral therapy.

## Author Contributions

LQ, TW, QT, GL, PW, and KC conceived and wrote the manuscript. LQ did the figure.

## Conflict of Interest Statement

The authors declare that the research was conducted in the absence of any commercial or financial relationships that could be construed as a potential conflict of interest.
